# Optimization of multiplex quantitative polymerase chain reaction based on response surface methodology and an artificial neural network-genetic algorithm approach

**DOI:** 10.1371/journal.pone.0200962

**Published:** 2018-07-25

**Authors:** Ping Pan, Weifeng Jin, Xiaohong Li, Yi Chen, Jiahui Jiang, Haitong Wan, Daojun Yu

**Affiliations:** 1 Hangzhou First People’s Hospital, Zhejiang Chinese Medical University, Hangzhou, Zhejiang, China; 2 College of Pharmaceutical Science, Zhejiang Chinese Medical University, Hangzhou, Zhejiang, China; 3 Zhejiang Hospital, Hangzhou, Zhejiang, China; 4 College of Life Science, Zhejiang Chinese Medical University, Hangzhou, Zhejiang, China; 5 Department of Clinical Laboratory, Affiliated Hangzhou First People’s Hospital, Zhejiang University School of Medicine, Hangzhou, Zhejiang, China; University of Helsinki, FINLAND

## Abstract

Multiplex quantitative polymerase chain reaction (qPCR) has found an increasing range of applications. The construction of a reliable and dynamic mathematical model for multiplex qPCR that analyzes the effects of interactions between variables is therefore especially important. This work aimed to analyze the effects of interactions between variables through response surface method (RSM) for uni- and multiplex qPCR, and further optimize the parameters by constructing two mathematical models via RSM and back-propagation neural network-genetic algorithm (BPNN-GA) respectively. The statistical analysis showed that Mg^2+^ was the most important factor for both uni- and multiplex qPCR. Dynamic models of uni- and multiplex qPCR could be constructed using both RSM and BPNN-GA methods. But RSM was better than BPNN-GA on prediction performance in terms of the mean absolute error (MAE), the mean square error (MSE) and the Coefficient of Determination (R^2^). Ultimately, optimal parameters of uni- and multiplex qPCR were determined by RSM.

## Introduction

Real-time quantitative PCR (qPCR) can quantitatively analyze a reaction template (nucleic acid) via the real-time continuous monitoring of the fluorescence signal generated from each cycle of the PCR amplification process. This technique has the advantages of being highly specific, highly sensitive, reproducible, accurately quantifiable, and highly automatable[1, 2]. Therefore, real-time PCR has been widely applied in fields such as molecular diagnostics, life sciences, agriculture, medicine, and food science[3–5]. Despite this broad application, there are still difficulties in the practical implementation of the technique, especially in multiplex qPCR systems. Due to the addition of multiple pairs of primers and probes, changes of factors such as annealing temperature, elongation temperature, and number of cycles can result in non-specific products and different effects on amplification. Furthermore, there is a lack of uniformity in data interpretation, and standardization needs to be improved[6]. These issues are mainly caused by the external factors that affect qPCR amplification and the practical difficulties in controlling these factors.

Prior studies[7–9]have attempted to optimize various qPCR parameters to ensure accuracy and stability. However, most of these studies employed single-factor tests when optimizing qPCR systems[9]. Although these studies determined the effects of individual factors on qPCR amplification, they required many and repeated tests and overlooked the effects of parameter interactions. The determined parameters may not have been optimized yet.

The recent studies to optimize qPCR parameters have focused on uniplex qPCR. The wide range of application for multiplex qPCR has brought increased attention to improving its amplification efficiency and optimizing the parameters. The construction of a reliable and dynamic mathematical model for multiplex qPCR that can be used to analyze the effects of interactions between various parameters is urgently needed. Response surface methodology (RSM) uses statistical and mathematical analysis to design experiments and involves a combination of experimental design techniques, including Plackett-Burman (PB) design, central composite design (CCD), Box-Behnken design, and others. RSM can evaluate the effects of variables on test results (yield) by analyzing experimental data. Additionally, RSM can analyze the interactions between various factors, and can be used to construct a mathematical model applicable for determining optimal conditions or ranges for a desired response[10]. RSM overcomes the disadvantages of single-factor tests, including the time required, the limited number of factors investigated, and the production of unreliable conclusions[11]. RSM has the advantages of being easy to implement and allowing investigation of the interplay of different factors. For these reasons, this methodology is commonly used in various fields such as pharmacy, architectural science, biology, agriculture, and microbiology[12–14].

As an alternative to RSM, artificial neural networks (ANNs)[15] are an integral component of artificial intelligence that can be applied for data analysis and prediction. As typical ANNs, back-propagation neural networks (BPNN) optimize and monitor the performance of neural networks under learning rules. BPNN can approximate any continuous function and have robust non-linear mapping capabilities. Genetic algorithms (GA) simulate the principle of survival of the fittest in nature and search for the most globally optimized combination of parameters for a given system. These algorithms can be used for discontinuous, indistinguishable, random, or highly nonlinear target functions. Therefore, the prediction models of ANNs are developed to analyze the obtained experimental data, while GA is utilized to optimize experimental parameters for the above well-established models. An increasing number of studies have focused on optimizing experimental conditions via ANNs and RSM in recent years[16–18]. However, there has been little work on the optimization of qPCR systems using these two methods. A few studies addressed that, as a predictive tool, ANNs can theoretically be used to understand complex systems and assist in optimizing conditions for biological experiments and related techniques such as PCR[19].

This study is based on previously developed AllGlo multiplex PCR systems for respiratory syncytial virus (RSV), influenza virus (INF), and human metapneumovirus (HMPV). CCD is used to design experiments with different concentrations of primers, probes, DNA polymerase, Mg^2+^, and dNTPs in uni- and multiplex qPCR systems, based on the experimental results, the interplay of the tested factors and their effects on cycle threshold (Ct) values of uni- and multiplex qPCR are analyzed and discussed by RSM. Subsequently, prediction models for the tested factors and Ct values are constructed via RSM and BPNN-GA respectively. The prediction performance of these two models is then evaluated using the coefficient of determination (R^2^), the mean absolute error (MAE), and the mean square error (MSE)[20–22]. The model resulting in better prediction performance is further tested for condition optimization. The optimal conditions for uni- and multiplex qPCR of the three viruses are then determined.

## Materials and methods

### Preparation of qPCR templates

*E*. *coli* DH5α samples containing RSV, INF, or HMPV target gene plasmids were cultured separately at 37°C under 5% CO_2_. A single colony was then transferred with an inoculation needle to Luria-Bertani (LB) culture and shaken overnight at 200 rpm/min at 37°C. Plasmid extraction was performed using a TaKaRa plasmid extraction kit following the manufacturer’s protocol (Takara Biomedical Technology Co., Ltd., Dalian, China, lot number 9760). The extracted plasmids were dissolved in 50 μL of eluent. A Nanodrop 2000 spectrophotometer was used to measure the A value of plasmid DNA at 260 nm/280 nm. Based on the measured copy number, the plasmids for the three viruses were separately diluted, mixed at equal ratios to 10^4^copies/mL, and stored at -20°C for later use. The repeated re-configuration of the template due to insufficient storage and material preparation could lead to biased results[23]. In this study, the amount of template needed was calculated and prepared prior to experiments, and the templates were stored in aliquots to increase test stability.

### Primers and probes

The genetic sequences of the three viruses were downloaded from the GenBank database. The primer picking tool and Oligo 6.22 from the NCBI database were used for comparison and optimization. Conservative segments with high homogeneity were selected for primer and probe designs. NCBI Blast was used to test the specificity of the primer and probe segments. Primers were synthesized by Intragen Trading (Shanghai) Co., Ltd., China, and probes were synthesized by Shanghai Yiyue Biotechnology Co., Ltd. (Table 1).

**Table 1:** Primers and probes used for AllGloqPCR.

Gene ID^a^	Primes and probes	Sequences(5’-3’)	Size(bp)
JX131645.1	RSV-F	GCACCGCCAAGACACTAGAA	179
	RSV-R	GTGGTTTGCCGAGGCTATGA	
	RSV-P	JUP(VIC)- GGA CCT GGG ACA CTC TCA ATC ATC T -JUP^b^	
KC731523.1	HMPV-F	GGGAGCAAAGCAGAAAGTTTGT	128
	HMPV-R	TTGCACAGACACATGCCCTA	
	HMPV-P	NEP(CY5)-GCT TAT GGA GCT GGT CAA ACA CTG C-NEP^b^	
L25072.1	INF-F	ACACCATCTGTGTGGGCTAC	136
	INF-R	CCGTTCAGACTGCAGAGCTT	
	INF-P	MAR(FAM)- CTC TAC AGA CAC TGT TGA CAC AGT ACT AG -MAR^b^	

^a^GenBank.

^b^JUP, NEP and MAR are three kinds of different AllGlo probe fluorochromes, and correspond to the currently used VIC, CY5 and FAM fluorescence, respectively[24].

### Experimental design

In this study, methods for uniplexqPCR are given in supplementary S1 Text. The concentrations of primer (Factor A), probe (Factor B), DNA polymerase (Factor C), Mg^2+^ (Factor D), and dNTPs (Factor E) were selected as independent variables and subjected to five levels of design using RSM-CCD. According to the recommended concentrations of these variables given by the VazymeLAmp® DNA Polymerase PCR kit (Vazyme Biotech Co., Ltd, Nanjing, China), baseline levels and ranges were confirmed. The coded values and actual values of the selected RSM design factors are provided in supplementary S1 Table. This study used a 50-test-point second-order RSM. The experiments for uni- and multiplex qPCR of the three viruses (RSV, INF, and HMPV) with the same designs; the uniplex qPCR designs are provided in S2 Table, and the multiplex qPCR designs are given in S3 Table.

### Quantitative PCR amplification

qPCR was performed in a final volume of 50 μL, which included 5 μL of 10 ×VazymeLAmp® Buffer (Mg^2+^-free), 0.5 μL of ROX Reference Dye II (50 ×) *^3^, and 4 μL of mix template. Additionally, MgCl_2_ (25 mmol/L), dNTP (10 mmol/L), primers (10 μmol/L), probes (10 μmol/L), and LAmp^TM^ DNA Polymerase (5 U/μL) were added in the concentrations provided in S2 and S3 Tables (VazymeLAmp® DNA Polymerase, Vazyme). An ABI 7500 Real-Time PCR System was used for amplification. A two-step method was implemented that included pre-denaturation at 95°C for 30 s, followed by 40 cycles of denaturation at 95°C for 5 s and elongation at 60°C for 32 s. Three parallel repeats were conducted for each test, and the averaged Ct value from the three repeats was taken as the result.

### RSM

**Fig 1:**
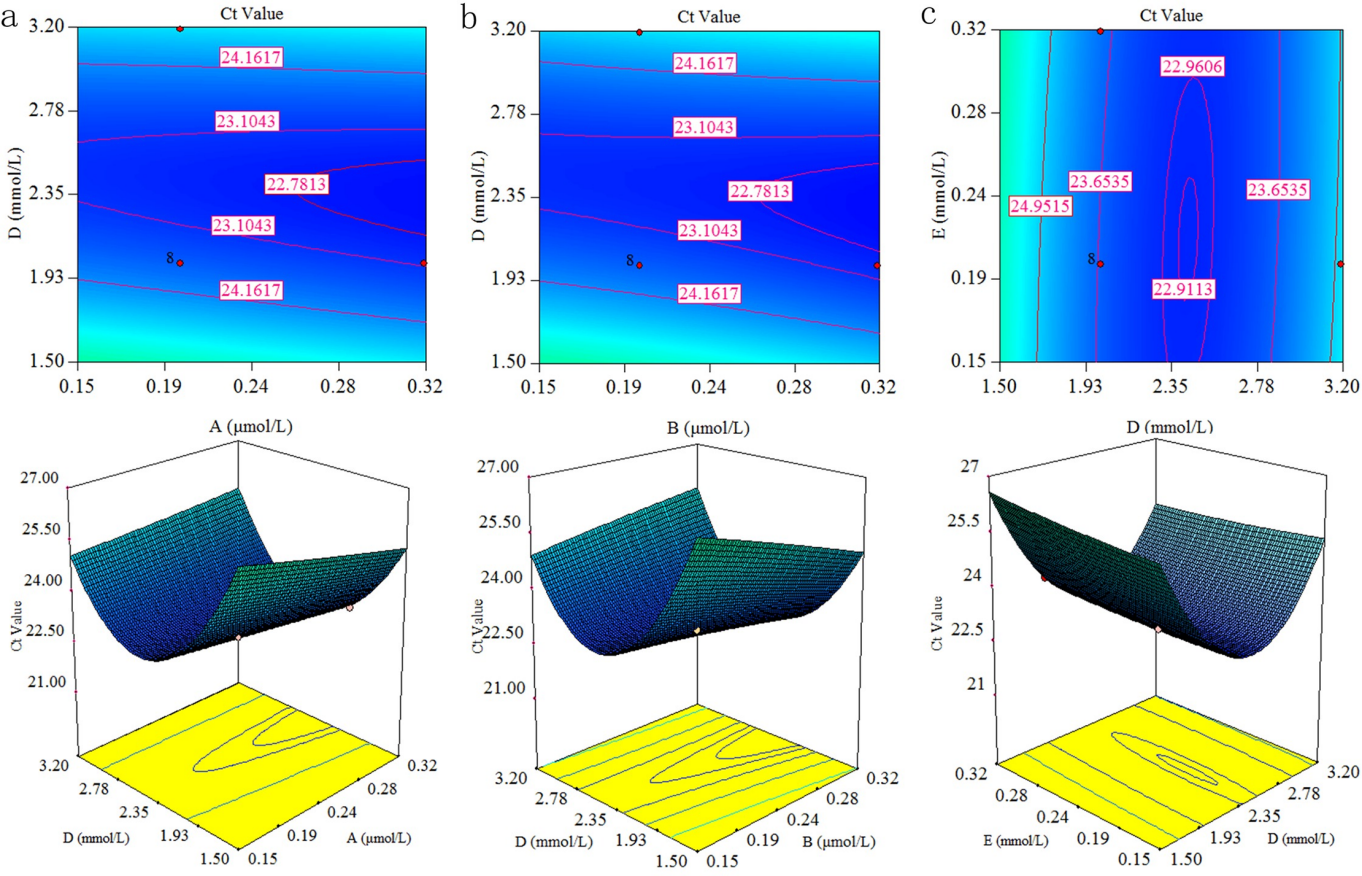
RSM-3D contour graphs showing groups of two interacting factors for multiplex qPCR of RSV. (A: primers, B: probes, D: Mg^2+^, E: dNTPs).a: The effects of interaction between primers and Mg^2+^ on the Ct value; b: The effects of the interaction between probes and Mg^2+^ on the Ct value; c: The effects of the interaction between Mg^2+^ and dNTPs on the Ct value. Graphs illustrate that the smaller Ct value can be obtained when the Mg^2+^ concentration is at a median level within the test range and the concentrations of primers and probes are higher, but the associated cost should be considered; in this study, the concentrations of these primers and probes are kept below 0.32 mmol/L.

**Fig 2:**
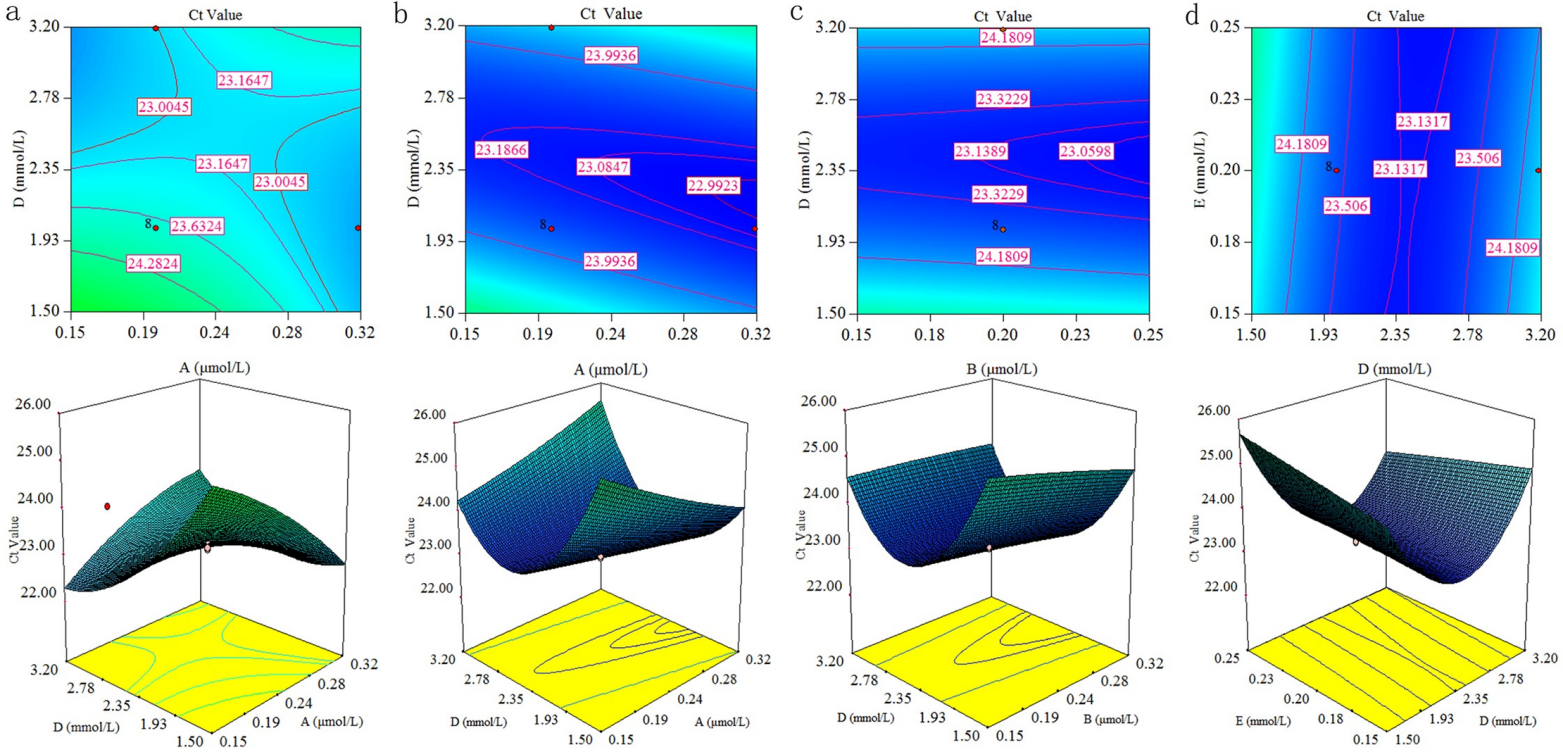
RSM-3D contour graph showing interactions between parameters for multiplex qPCR of INF and HMPV. (A: primers, B: probe, D: Mg^2+^, E: dNTPs)a: The effects of the interaction between Mg^2+^and primers on the Ct value for multiplex qPCR of INF; b: The effects of interaction between primers and Mg^2+^ on the Ct value for multiplex qPCR of HMPV; c: The effects of the interaction between probes and Mg^2+^ on the Ct value multiplex qPCR of HMPV; d: The effects of the interaction between Mg^2+^and dNTPs on the Ct value for multiplex qPCR of HMPV.

The averaged Ct value resulting from the three parallel tests was treated as the response value (*Y*). Design-Expert.V8.0.6 was used for RSM analysis of the test data. The statistical significance of the RSM-based model (model I) was checked by analysis of variance (ANOVA). The variables were treated as continuous random factors. The complete CCD matrix for the experimental Ct values of uni- and multiplex qPCR is described in S2 and S3 Tables. Optimized conditions for Ct values were obtained in combination with 3D response surface graphs (Fig 1 and Fig 2, S1 and S2 Figs).

The mathematical expression used by model I to describe the response value, *Y* (Ct value), versus the five factors studied (A, B, C, D, and E) can be written as follows[25]:

$$Y=\beta_{0}+\sum_{i=1}^{k}{\beta_{i}X}_{i}+\sum_{i=1}^{k}\beta_{ii}X_{i}^{2}+\sum_{i=1}^{k}\sum_{j=1}^{k}\beta_{ij}X_{i}X_{j}+\varepsilon$$ (1)

*Y* is the response value (Ct value), *X*_*i*_ and *X*_*j*_ denote the coded levels of the independent variables, and *β*_*0*_ is the intercept; *i*, *j*, *β*_*i*_, *β*_*j*_, and *β*_*ij*_ are coefficients, *ε* is the test error, and *k* represents the number of independent factors. In the following descriptions, *Y*_*1*_, *Y*_*2*_, and *Y*_*3*_ represent the corresponding multiplex qPCR RSM polynomial equations for RSV, INF, and HMPV, respectively.

### BPNN-GA

A 3-layer BPNN-GA comprised of input, hidden layer, and output layers was used to construct a mathematical model (model II), Concentrations of primers, probes, DNA polymerase, Mg^2+^, and dNTPs were used as the 5 inputs, and the Ct value was the output. Library (neuralnet) of R language was used for analysis, and we scaled Ct values to lie within the range (-)2.378-(+)2.378 in BPNN-GA, while the activation functions were sigmoid for all the models, a five-fold cross-validation was employed to select the number of nodes on the hidden layer and to train the model[26].

Optimization of uni- and multiplex qPCR conditions for the three viruses (RSV, INF, and HMPV) was performed using the trained model obtained using five-fold cross-validation with GA. Optimized conditions were obtained along with the corresponding predictive Ct values.

### Model evaluation and validation

The Coefficient of Determination (R^2^), the mean absolute error (MAE) and the mean square error (MSE) were monitored to evaluate and compare the stabilities and prediction performances of the model I and model II. R^2^ is applicable to the training data set, and the larger R^2^ indicates that the more percent of the variance in the response variable can be explained by the explanatory variables[27]. And the smaller the MAE and MSE means the better the model[28]. The model that performed better was selected as a predictive model for condition optimization. Validation was conducted under the predictive optimal operation conditions subsequently.

The equations representing the evaluation indices used for model performance are provided below[27]:

$$R^{2}=1-\frac{\sum_{i=1}^{n}(Y_{i,p}-Y_{i,e}{)}^{2}}{\sum_{i=1}^{n}(Y_{i,e}-Y_{a}{)}^{2}}$$ (2)

$$MAE=\frac{1}{n}\sum_{i=1}^{n}\left|Y_{i,p}-Y_{i,e}\right|$$ (3)

$$MSE=\frac{\sum_{i=1}^{n}{\left(Y_{i,p}-Y_{i,e}\right)}^{2}}{n}$$ (4)

*Y*_*i*,*p*_ is the value predicted by the model, *Y*_*i*,*e*_ is the experimental Ct value, *Y*_*a*_ is the averaged experimental Ct value, and n is the number of data points.

## Results

### RSM

The effects of operating variables were investigated according to the statistical analysis of CCD. According to variance analysis (Table 2), the *F* and *P* values of the corresponding multiplex qPCR models for the three viruses were *F* = 6.61 and *P*<0.0001, *F* = 3.67 and *P* = 0.0009, and *F* = 7.89 and *P*<0.0001; the *P* values were all less than 0.05, indicating that all the RSM models of multiples qPCR were statistically significant; meanwhile, the statistical results on factors showed that there was evident that at least one of the 5 predictors had an effect on the response, such as the statistical results on factors of RSV, the *P* value of DNA polymerase and Mg^2+^ were 0.0452 and <0.0001 respectively, demonstrated DNA polymerase and Mg^2+^were statistically significant for Ct value in multiplex qPCR of RSV, in addition, the *P* value of primers, probes, and other factors were >0.5, they had no significant effect on Ct value; the statistical results on factors of INF and HMPV showed the same conclusion. Variance analysis of uniplex qPCR was given in S4 Table.

**Table 2:** Analysis of variance (ANOVA) for the Ct values of multiplex qPCR using RSM-CCD.

Source	*Df*^b^	RSV^a^			INF^a^			HMPV^a^		
		*SS*^b^	*F*^b^	*P*	*SS*^b^	*F*^b^	*P*	*SS*^b^	*F*^b^	*P*
Model	20	195.630	6.610	<0.0001***	89.860	3.670	0.0009**	90.320	7.890	<0.0001***
A	1	2.48	2.02	0.1834	3.66	2.99	0.0949	4.30	7.52	0.0105*
B	1	2.83	3.35	0.1561	2.16	1.76	0.1950	0.98	1.71	0.2017
C	1	5.86	5.89	0.0452*	3.97	3.24	0.0828	0.011	0.019	0.8904
D	1	114.79	82.64	<0.0001***	33.38	27.23	<0.0001***	51.94	90.75	<0.0001***
E	1	0.094	0.024	0.7922	6.08	4.96	0.0342*	1.10	1.93	0.1756
AB	1	1.47	1.07	0.3022	6.71	5.48	0.0266*	0.15	0.26	0.6127
AC	1	1.92	1.45	0.2402	3.84	3.13	0.0878	4.28	7.47	0.0107*
AD	1	0.70	0.44	0.4736	4.38	3.58	0.0690	2.36	4.13	0.0518
AE	1	0.92	0.61	0.4143	1.56	1.27	0.2687	0.12	0.21	0.6496
BC	1	0.17	0.34	0.7272	0.95	0.78	0.3853	0.76	1.33	0.2578
BD	1	1.36	1.79	0.3214	0.70	0.57	0.4547	0.094	0.16	0.6886
BE	1	5.16	4.36	0.0592	10.72	8.75	0.0062**	4.489×10^−3^	7.844×10−^3^	0.9301
CD	1	0.63	0.38	0.4968	2.52	2.06	0.1628	0.28	0.50	0.4869
CE	1	3.55	4.22	0.1143	1.80	1.47	0.2361	0.063	0.11	0.7430
DE	1	0.15	0.042	0.7429	2.95	2.40	0.1322	1.48	2.58	0.1196
A^2^	1	2.808×10^−3^	0.088	0.9637	0.49	0.40	0.5328	0.061	0.11	0.7458
B^2^	1	6.068×10^−3^	0.028	0.9467	1.405×10^−7^	1.146×10^−7^	0.9997	5.920×10^−3^	0.010	0.9197
C^2^	1	1.23	2.30	0.3460	0.94	0.77	0.3877	0.022	0.038	0.8460
D^2^	1	49.71	21.76	<0.0001***	3.08	2.51	0.1243	21.32	37.25	<0.0001***
E^2^	1	0.028	0.17	0.8866	0.11	0.088	0.7695	8.105×10^−3^	0.014	0.9061
Residual	28	37.370			34.320			16.020		
Lack of Fit	21	37.320	277.800	<0.0001***	34.220	113.830	<0.0001***	15.960	84.010	<0.0001***
Pure Error	7	0.0450			0.100			0.063		
Cor Total	48	233.000			124.190			106.340		
Adequate pression		14.275			8.9560			14.392		

*p−value <0.05

**p−value <0.01

***p−value <0.001.

^a^RSV, HMPV, INF are three virus used in this study.

^b^*Df*: Degree of freedom; *SS*: Sum of Squares; *F*, *F*-value.

$$Y_{1}=23.600-0.420\times C-1.630\times D+0.950\times D^{2}$$ (5)

$$Y_{2}=23.740-0.880\times D+0.370\times E-0.460\times AB+0.580\times BE$$ (6)

$$Y_{3}=23.600-0.320\times A-1.100\times D+0.370\times AC+0.620\times D^{2}$$ (7)

Eqs (5)–(7) represent the multiplex qPCR polynomial model for the three viruses (RSV, INF and HMPV). Coefficients that had no statistical significance are eliminated. As seen from polynomial equations of multiplex qPCR for three viruses, the Mg^2+^ all affected significantly the Ct values, thus, the most important factor under the conditions used for these experiments was the concentration of Mg^2+^.

The RSM-3D plot used for RSV multiplex qPCR is illustrated in Fig 1, and the RSM-3D plot other viruses of multiplex qPCR is set out in Fig 2. The x- and y-axes represent the two interacting factors, and the obtained Ct value is expressed on the z-axis. The color, height, and contour of the curves indicate different response levels. Interacting factors are described as AD, BD, and DE. As can be seen from Figures, Mg^2+^ is the most important parameter influencing the Ct value. The RSM-3D plots used for uniplexqPCR are shown in S1 and S2 Figs.

### BPNN-GA

The fitting error and predictiveerrorof five-fold cross-validation of model II for multiplex qPCR produced by training neurons in the hidden layer are provided in Table 3. The fitting error and predictive error are the bases used to select the number of nodes in the hidden layer. When fitting error and predictive error were relatively small, and took the minimum prediction error as the primary condition, the corresponding number of hidden-layer nodes was treated as the training model. Such as RSV multiplex qPCR, the fitting error had improvement in performance by increasing the number of hidden layer, whereas, the performance of predictive error showed negative effect for 3, 5 neurons, respectively, so the number of neurous was 4 as final model of RSV multiplex qPCR. At the same, the number of nodes selected for INF and HMPV multiplex qPCR was 4, 3, respectively. And the fitting error and predictive error of five-fold cross-validation of model II for uniplex qPCR were given in S5 Table.

**Table 3:** Five-fold cross-validation of model II for multiplex qPCR.

Neurons	RSV^a^		INF^a^		HMPV^a^	
	Error of fit ($\bar{\chi }\pm s$)	Error of prediction ($\bar{\chi }\pm s$)	Error of fit ($\bar{\chi }\pm s$)	Error of prediction ($\bar{\chi }\pm s$)	Error of fit ($\bar{\chi }\pm s$)	Error of prediction ($\bar{\chi }\pm s$)
1	0.099±0.063	0.048±0.023	0.120±0.098	0.075±0.132	0.079±0.12	0.057±0.082
2	0.056±0.058	0.055±0.023	0.077±0.089	0.079±0.077	0.040±0.078	0.022±0.069
3	0.035±0.035	0.039±0.023	0.056±0.072	0.043±0.080	0.023±0.055	0.016±0.044
4	0.042±0.049	0.034±0.041	0.051±0.079	0.042±0.059	0.022±0.024	0.020±0.052
5	0.031±0.050	0.040±0.049	0.046±0.064	0.046±0.06	0.024±0.058	0.016±0.049

^a^RSV, HMPV, INF are three virus used in this study.

### RSM versus BPNN-GA

In this study, RSM and BPNN-GA were used for data analysis. Corresponding mathematical models were constructed and referred to as model I (RSM) and model II (BPNN-GA).

The R^2^ values for model II were closer to 1 than for model I in both uni- and multiplex PCR for all three viruses, The MAE and MSE of model II was much smaller than those of model Ifor both uni- and multiplex qPCR of the three viruses (Table 4, S6 Table). Though R^2^ of model II are all larger than RSM, compared with RSM, ANN's predictive Ct values are more deviated from the actual situation, especially in HMPV and INF for multiplex PCR (Table 5, S7 Table), therefore, we believe that in the PCR system optimization, RSM is more appropriate than ANN. Hence, model I was selected as the final prediction model.

**Table 4:** Performance numbers of BPNN-GA versus RSM models for multiplex qPCR.

parameter	RSV^a^		INF^a^		HMPV^a^	
	Model I	Model II	Model I	Model II	Model I	Model II
R^2^	0.847	0.980	0.746	0.940	0.864	0.976
MAE^b^	0.633	0.0007	0.613	0.0009	0.483	0.0003
MSE^b^	0.762	0.0015	0.700	0.0021	0.327	0.0008

^a^RSV, HMPV, INF are three virus used in this study.

^b^MAE means the mean absolute error; MSE means the mean square error.

**Table 5:** Optimization conditions and predictive Ct value of model I and model II for multiplex qPCR.

Factors	RSV^b^		INF^b^		HMPV^b^	
	Model I	Model II	Model I	Model II	Model I	Model II
A^a^(μM)	0.290	0.319	0.230	0.319	0.320	0.164
B^a^(μM)	0.310	0.319	0.260	0.319	0.320	0.320
C^a^(U^c^/μL)	0.040	0.081	0.050	0.319	0.030	0.081
D^a^(mM)	2.550	0.172	2.350	0.319	0.800	0.195
E^a^(mM)	0.160	0.319	0.090	0.144	0.080	0.082
Predictive Ct value	21.485	8.3974	19.908	17.4232	24.362	-9.5411

^a^A: primers, B: probes, C: DNA polymerase, D: Mg^2+^, E: dNTPs.

^b^RSV, HMPV, INF are three virus used in this study.

^c^U: active unit of enzyme.

### Validation

After comparing the stabilities and prediction performances of models I and II, model I was selected for condition optimization. The optimized conditions were subjected to validation tests, three parallel repeats were performed and relative errors (shown as Eq ((8)) between validated Ct values and predictive Ct values for multiplex qPCR for RSV, INF and HMPV are 3.351%, 13.744%, 4.550% respectively. The relative errors of the validated Ct values of uniplex qPCR for RSV, INF and HMPV are 6.748%, 1.520%, 4.590% respectively. The validation results indicate a greater relative error for multiplex qPCR of INF; the error rates for multiplex qPCR of the other two viruses were within an acceptable range.

$$Relative\;Error=\frac{\left|{Ct}_{p}-{Ct}_{e}\right|}{{Ct}_{e}}\times 100\%$$ (8)

Relative Error: Actual relative error, generally given as a percentage; Ct_*p*_: predictive Ct value under optimal conditions; Ct_*e*_: experimental Ct value under optimal conditions

## Discussion

As a detection technique, qPCR has significant advantages[1]. However, the number of factors influencing the process is relatively high, and the interactions between these factors are complicated, especially in multiplex qPCR systems. Due to the addition of multiple pairs of primers and probes, changes to factors such as annealing temperature, elongation temperature, and number of cycles can result in non-specific products and different effects on amplification, which leads to difficulties in comparing results of qPCR.

Considering the important effects of different primers on uni- and multiplex qPCR[29], we considered the primer melting temperature (*T*_*m*_) and GC percentage as well as the homogeneity of the primers and the target nucleic acid sequence when designing primers. Having close *T*_*m*_values can improve the amplification of product in multiplex qPCR[30–32]. The uni- and multiplex qPCR mathematical models built using RSM for the three viruses produced statistically significant results. Additionally, adequate precision measures the signal to noise ratio, and this ratio greater than 4 is desirable. The ratios for the models were all greater than 4 (Table 2, S4 Table), indicating models are sufficiently precisionand can be used to guide experiments[33].

As seen from Polynomial equations of uni- and multiplex qPCR for three viruses, the Mg^2+^ all significantly affected the Ct value. Meanwhile, Fig 1 also indicate magnesium ion is the most important parameter influencing the Ct value. Thus, the most important factor under the conditions used for these experiments is the concentration of magnesium ion.

To our knowledge[34], dNTP and DNA polymerase are competing reagents in PCR systems. Free magnesium is necessary for DNA polymerase to exert its biological activity; the formation of bonds between dNTP and DNA also requires magnesium ion. Furthermore, in multiplex PCR with a constant dNTP concentration, the amplification product increases as the concentration of Mg^2+^ increases, and non-specific bands are eliminated. However, when an excessive concentration of Mg^2+^ is used, amplification slows down and may even stop, which is speculated to result from a suppression effect[35].

Multiple factors affecting multiplex PCR amplification are consistent with those observed in this study. Mg^2+^ significantly influenced the formation of the PCR amplification product; at appropriate concentrations, positive effects on the amount of PCR product were observed, but suppression occurred at excessively high concentrations. Under the experimental conditions used, changes in dNTP concentration did not significantly affect PCR amplification, which could be due to the range of dNTP concentration was too narrow in this study.

Interestingly, Mg^2+^ had a positive effect on uniplex PCR but a negative effect on multiplex qPCR of RSV in this study, which are concordant with prior work[32], DNase concentration differentially affects the amplification of uni- and multiplex PCR; an increase in DNase concentration in uniplex PCR leads to an increase in non-specific product but positively affects multiplex PCR amplification.

Nonetheless, model I was relatively better than model II under the experimental conditions used in this study, its prediction function was still unsatisfactory. This could be related to the limitations of RSM, such as the ability to only build quadratic polynomial functions[36] and the inability to portray the relationship between multiplex qPCR factors. Although model II was better at solving non-linear functions[37], it produced over-fittings[28].

As a result, model I was selected for condition optimization. However, in multiplex qPCR, unlike the concentrations of template-specific primers and probes, the same concentrations must be used for DNA polymerase, Mg^2+^, and dNTPs. Therefore, further adjustments to multiplex qPCR conditions based on parameter optimization results are necessary. For multiplex qPCR, combined with RSM-3D plots, the smaller Ct values were obtained at greater concentrations of primers and probes. However, considering the increase in non-specificity when primer and probe concentrations are too high and the differences in the optimized conditions for the primers used for different viruses[32], the primers and probes concentrations suggested by the RSM models for each virus were determined to be the optimized condition for each virus. DNA polymerase and Mg^2+^ behaved similarly within a median range of concentrations (0.04–0.08 mmol/L and 2.0–2.5 μmol/L respectively), which could be sufficient for all three viruses. Considering the increase in non-specificity triggered by a high concentration of Mg^2+^[29], the concentration used was set at 2.35 mmol/L. dNTPs did not significantly influence the response value. The final and cost-effective optimal conditions to be used for uni- and multiplex qPCR are given in Table 6 and S8 Table.

**Table 6:** Optimal conditions for multiplex qPCR.

Factors	RSV^b^	INF^b^	HMPV^b^
A^a^(μmol/L)	0.290	0.230	0.320
B^a^(μmol/L)	0.310	0.260	0.320
C^a^(U^c^/μL)	0.050	0.050	0.050
D^a^(mmol/L)	2.350	2.350	2.350
E^a^(mmol/L)	0.160	0.160	0.160

^a^A: primers, B: probes, C: DNA polymerase, D: Mg^2+^, E: dNTPs.

^b^RSV, HMPV, INF are three virus used in this study.

^c^U:active unit of enzyme.

## Conclusions

Optimization conditions of uni- and multiplex qPCR were predicted by RSM. By operating designed experiments, the effects between different variations were investigated. Furthermore, two mathematical models were constructed via RSM and BPNN-GA. The statistical results of RSM indicated that Mg^2+^ was the most important factor in this study. And interestingly, the same factor played different role in different reaction system. Though the R^2^of GA-BPNNs are all larger than RSM, the predictive Ct value of qPCR is too different from the actual value, therefore, we believe that in the PCR system optimization, RSM is more appropriate than ANN, perhaps because its internal relationship, so RSM was performed better in modeling both uni- and multiplex qPCR.

## Supporting information

S1 Fig RSM-3D contour graph showing interactions between parameters for uniplex qPCR of RSV. (A: primers, B: probe, D: Mg^2+^, E: dNTPs) a: The effects of interaction between primers and Mg^2+^ on the Ct value; b: The effects of the interaction between probes and Mg^2+^on the Ct value; c: The effects of the interaction between Mg^2+^and dNTPs on the Ct value.
(TIF)

S2 Fig RSM-3D contour graph showing interactions between parameters for uniplexqPCR of INF and HMPV. (A: primers, B: probe, C: DNA polymerase, D: Mg^2+^)a: The effects of interaction between primers and Mg^2+^ on the Ct value for uniplex qPCR of INF; b: The effects of the interaction between probes and Mg^2+^ on the Ct value for uniplex qPCR of INF; c: The effects of the interaction between Mg^2+^and DNA polymerase on the Ct value for uniplex qPCR of INF; d: The effects of the interaction between Mg^2+^and DNA polymerase on the Ct value for uniplex qPCR of HMPV.
(TIF)

S1 Table Independent variables and levels used for response surface design. (PDF)

S2 Table CCD matrix for the independent variables and experimental results from uniplexqPCR. (PDF)

S3 Table CCD matrix for the independent variables and experimental results from multiplex qPCR. (PDF)

S4 Table Analysis of variance (ANOVA) for the Ct values of uniplex qPCR using RSM-CCD. (PDF)

S5 Table Five-fold cross-validation of Ct values for uniplex qPCR. (PDF)

S6 Table Performance numbers of BPNN-GA versus RSM models for uniplex qPCR. (PDF)

S7 Table Optimization conditions and predictive Ct value of model I and model II for uniplex qPCR. (PDF)

S8 Table Optimal conditions for uniplex qPCR. (PDF)

S1 Text Results of uniplexqPCR. (PDF)
