# Supplementary figures and images for: Optimization of multiplex quantitative polymerase chain reaction based on response surface methodology and an artificial neural network-genetic algorithm approach
Source: PLoS One. 2018 Jul 25;13(7):e0200962. doi: 10.1371/journal.pone.0200962 (PMC6059488; doi:10.1371/journal.pone.0200962)

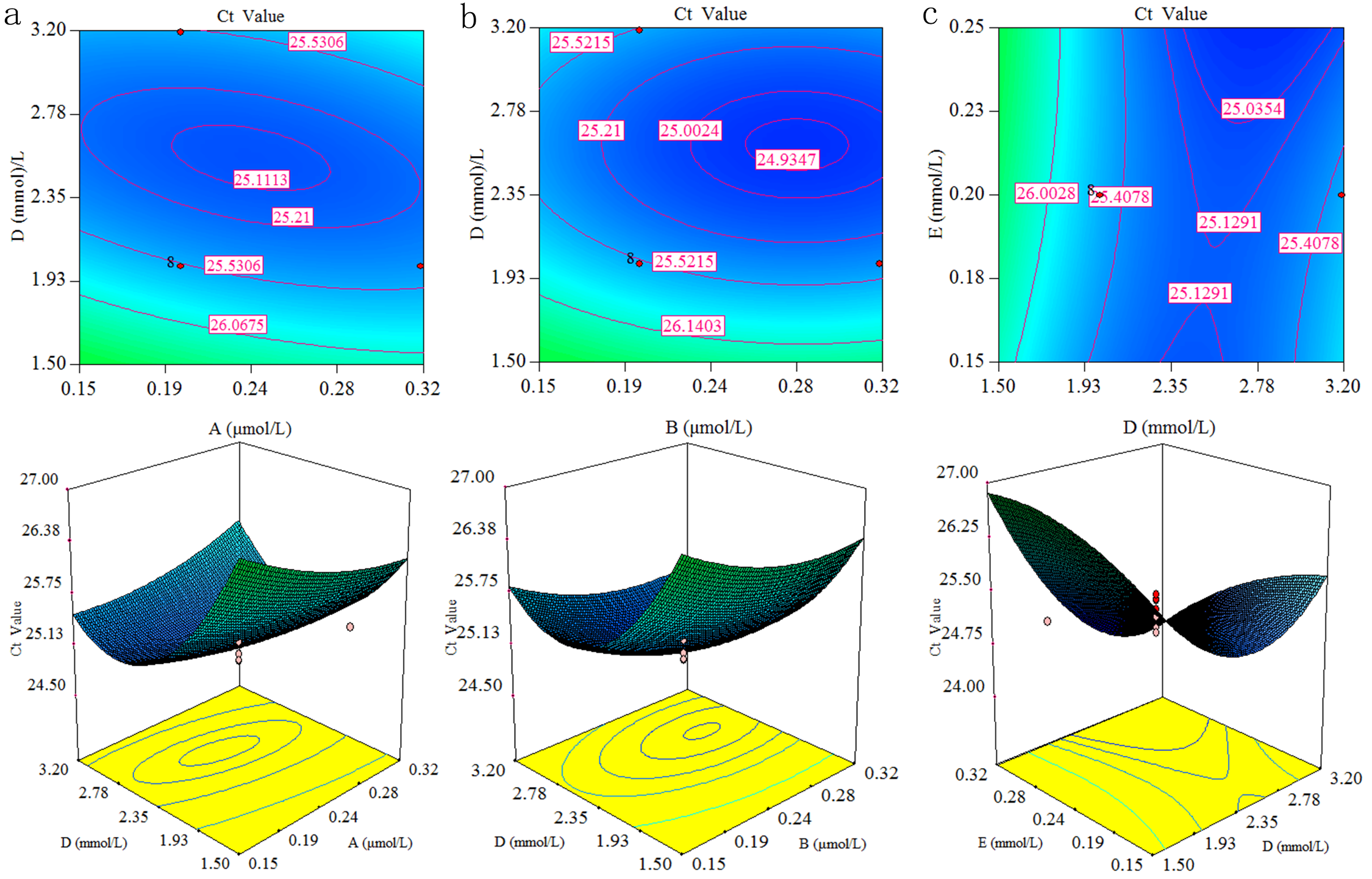

Supplement: S1 Fig — (A: primers, B: probe, D: Mg2+, E: dNTPs) a: The effects of interaction between primers and Mg2+ on the Ct value; b: The effects of the interaction between probes and Mg2+on the Ct value; c: The effects of the interaction between Mg2+and dNTPs on the Ct value. (TIF) [file pone.0200962.s001.tif]

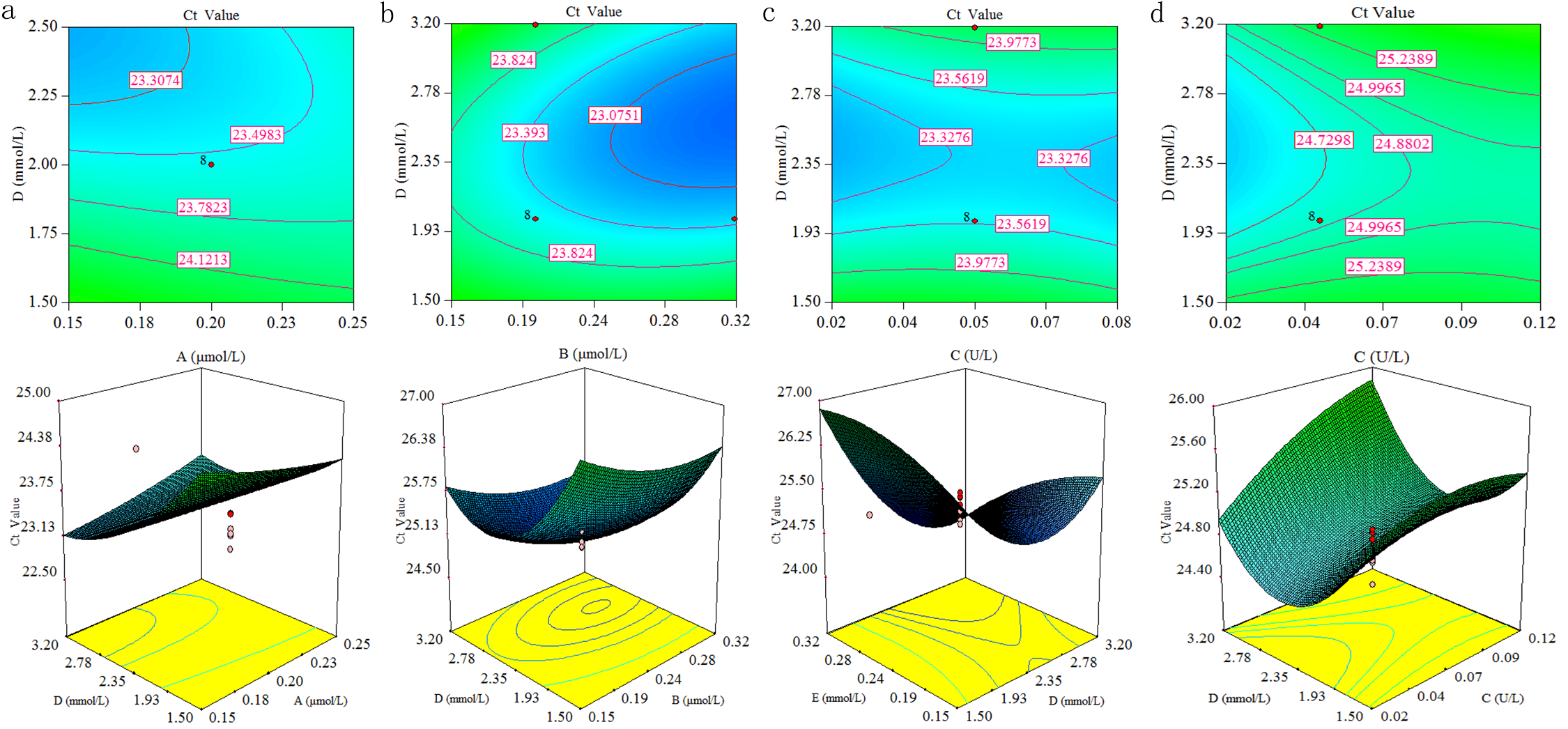

Supplement: S2 Fig — (A: primers, B: probe, C: DNA polymerase, D: Mg2+)a: The effects of interaction between primers and Mg2+ on the Ct value for uniplex qPCR of INF; b: The effects of the interaction between probes and Mg2+ on the Ct value for uniplex qPCR of INF; c: The effects of the interaction between Mg2+and DNA polymerase on the Ct value for uniplex qPCR of INF; d: The effects of the interaction between Mg2+and DNA polymerase on the Ct value for uniplex qPCR of HMPV. (TIF) [file pone.0200962.s002.tif]
