# Supplementary material for: Optimization of multiplex quantitative polymerase chain reaction based on response surface methodology and an artificial neural network-genetic algorithm approach
Source: PLoS One. 2018 Jul 25;13(7):e0200962. doi: 10.1371/journal.pone.0200962 (PMC6059488; doi:10.1371/journal.pone.0200962)
Supplement: S1 Table — (PDF) [file pone.0200962.s003.pdf]

S1 Table. Independent variables and levels used for response surface design

Factors	Symbol（unit）	Level				
		Lowest(-2.378)	Low(-1)	Central(0)	High(+1)	Highest(+2.378)
Primers	A[a](μmol/L)	0.080	0.150	0.200	0.250	0.320
Probe	B[a](μmol/L)	0.080	0.150	0.200	0.250	0.320
LAmp™ DNA Polymerase	C[a](U[b]/μL)	-0.020	0.020	0.050	0.080	0.120
magnesium ion	D[a](mmol/L)	0.800	1.500	2.000	2.500	3.200
dNTP	E[a](mmol/L）	0.080	0.150	0.200	0.250	0.320

[a]A: primers, B: probe, C: DNA polymerase, D: Mg^{2+}, E: dNTPs.

[b]U: active unit of enzyme.
