# Supplementary material for: Optimization of multiplex quantitative polymerase chain reaction based on response surface methodology and an artificial neural network-genetic algorithm approach
Source: PLoS One. 2018 Jul 25;13(7):e0200962. doi: 10.1371/journal.pone.0200962 (PMC6059488; doi:10.1371/journal.pone.0200962)
Supplement: S2 Table — (PDF) [file pone.0200962.s004.pdf]

S2Table.CCD matrix for the independent variables and experimental results from uniplexqPCR

Run order	Code of variables					Response(Ct value)[c]		
	A[a]	B[a]	C[a]	D[a]	E[a]	RSV[b]	INF[b]	HMPV[b]
1	1	1	1	1	1	24.826±0.250	23.107±0.025	24.175±0.074
2	0	0	0	0	0	25.826±0.145	24.107±0.082	24.580±0.099
3	1	-1	-1	1	-1	25.282±0.094	23.765±0.021	23.613±0.190
4	0	0	0	0	0	25.534±0.199	23.590±0.105	24.999±0.231
5	1	1	-1	-1	-1	26.554±0.183	24.262±0.240	24.768±0.020
6	-1	1	-1	-1	-1	25.590±0.142	23.509±0.136	25.356±0.196
7	0	0	0	0	0	25.634±0.246	23.296±0.023	24.816±0.124
8	-1	-1	-1	1	1	25.585±0.139	23.316±0.209	24.748±0.165
9	-1	1	-1	-1	1	27.321±0.167	25.397±0.041	25.165±0.287
10	1	-1	1	1	-1	25.351±0.079	23.736±0.207	25.066±0.221
11	-1	1	1	1	-1	24.694±0.006	22.496±0.214	24.882±0.142
12	1	-1	1	-1	1	26.871±0.197	24.930±0.072	25.174±0.054
13	-1	1	-1	1	-1	24.846±0.165	22.723±0.226	24.770±0.079
14	0	0	0	0	0	25.315±0.130	23.382±0.010	25.056±0.014
15	0	-2.378	0	0	0	26.553±0.193	24.301±0.173	25.296±0.082
16	-1	-1	1	1	1	25.359±0.119	23.494±0.203	25.190±0.058
17	1	-1	-1	1	1	25.157±0.113	23.531±0.087	24.597±0.113
18	1	1	1	1	-1	24.959±0.306	23.081±0.156	24.660±0.045
19	1	1	-1	1	1	24.851±0.262	22.852±0.327	24.284±0.173
20	1	-1	1	-1	-1	26.231±0.257	24.090±0.123	25.416±0.017
21	-1	-1	1	-1	1	27.341±0.076	24.659±0.171	25.608±0.201
22	1	1	1	-1	-1	25.125±0.317	23.434±0.099	24.575±0.173
23	0	0	0	-2.378	0	29.553±0.254	26.624±0.167	27.662±0.017
24	2.37	0	0	0	0	25.110±0.227	23.398±0.113	24.297±0.201
25	1	1	-1	-1	1	26.320±0.099	24.037±0.048	24.448±0.049
26	-1	-1	1	-1	-1	26.734±0.142	24.089±0.037	25.658±0.098
27	0	0	0	0	0	25.392±0.062	23.101±0.011	25.088±0.084
28	0	0	0	0	0	25.321±0.194	23.316±0.277	24.804±0.013
29	-1	-1	-1	-1	1	27.853±0.067	25.211±0.104	26.937±0127
30	0	0	-2.3	0	0	/	/	/
31	-1	-1	-1	1	-1	25.610±0.111	23.687±0.083	24.813±0.056
32	-1	1	1	-1	-1	26.343±0.160	24.392±0.110	25.314±0.097
33	0	0	0	0	0	25.753±0.047	23.596±0.095	24.810±0.181
34	1	-1	-1	-1	-1	26.157±0.252	24.922±0.053	24.751±0.106
35	0	0	0	0	-2.378	25.468±0.201	23.707±0.134	24.939±0.003

36	0	0	0	0	0	25.770±0.096	23.930±0.084	24.787±0.089
37	0	2.378	0	0	0	25.620±0.167	23.568±0.109	24.769±0.028
38	-1	1	1	1	1	24.970±0.164	22.938±0.063	24.879±0.036
39	0	0	0	0	2.378	24.843±0.140	23.235±0.037	24.760±0.093
40	1	-1	1	1	1	26.014±0.157	25.571±0.124	25.335±0.085
41	-1	-1	-1	-1	-1	26.513±0.128	24.853±0.042	24.985±0.102
42	1	-1	-1	-1	1	27.030±0.078	23.855±0.066	25.002±0.069
43	0	0	2.37	0	0	25.845±0.043	22.785±0.088	25.174±0.090
44	-1	1	-1	1	1	25.398±0.042	22.437±0.103	24.589±0.034
45	-1	1	1	-1	1	26.732±0.118	24.162±0.110	25.791±0.172
46	0	0	0	2.378	0	25.105±0.010	24.057±0.206	24.873±0.048
47	1	1	1	-1	1	26.361±0.148	24.373±0.182	25.181±0.116
48	1	1	-1	1	-1	25.307±0.036	24.079±0.189	24.508±0.076
49	-1	-1	1	1	-1	25.747±0.056	23.247±0.054	25.352±0.194
50	-2.3	0	0	0	0	26.782±0.046	24.254±0.182	25.528±0.085

[a]A: primers, B: probe, C: DNA polymerase, D: Mg^{2+}, E: dNTPs.

[b]RSV、HMPV、INF are three virus used in this study.

[c]The results of Ct value in table are expressed as $\bar{\chi}$±s, and n=3.
