# Supplementary material for: Optimization of multiplex quantitative polymerase chain reaction based on response surface methodology and an artificial neural network-genetic algorithm approach
Source: PLoS One. 2018 Jul 25;13(7):e0200962. doi: 10.1371/journal.pone.0200962 (PMC6059488; doi:10.1371/journal.pone.0200962)
Supplement: S3 Table — (PDF) [file pone.0200962.s005.pdf]

S3Table. CCD matrix for the independent variables and experimental results from multiplex qPCR

Run order	Code of variables					Response(Ct value)[c]		
	A[a]	B[a]	C[a]	D[a]	E[a]	RSV[b]	INF[b]	HMPV[b]
1	1	1	1	1	1	23.068±0.228	23.224±0.217	23.127±0.077
2	0	0	0	0	0	23.624±0.090	23.598±0.165	23.544±0.082
3	1	-1	-1	1	-1	23.881±0.198	23.515±0.238	23.344±0.134
4	0	0	0	0	0	23.568±0.074	23.852±0.047	23.543±0.044
5	1	1	-1	-1	-1	23.948±0.123	23.193±0.071	23.759±0.192
6	-1	1	-1	-1	-1	26.164±0.277	24.823±0.201	25.327±0.150
7	0	0	0	0	0	23.535±0.136	23.735±0.167	23.653±0.109
8	-1	-1	-1	1	1	23.445±0.148	23.114±0.027	23.305±0.055
9	-1	1	-1	-1	1	29.336±0.080	30.628±0.125	28.352±0.150
10	1	-1	1	1	-1	22.731±0.090	23.880±0.134	23.233±0.053
11	-1	1	1	1	-1	22.494±0.091	21.205±0.119	23.189±0.072
12	1	-1	1	-1	1	27.079±0.104	27.047±0.180	27.542±0.291
13	-1	1	-1	1	-1	22.753±0.205	22.601±0.093	23.085±0.017
14	0	0	0	0	0	23.589±0.049	23.814±0.060	23.783±0.061
15	0	-2.37	0	0	0	24.012±0.168	24.287±0.069	23.968±0.075
16	-1	-1	1	1	1	22.648±0.052	22.177±0.083	23.272±0.013
17	1	-1	-1	1	1	22.906±0.144	22.916±0.132	22.987±0.105
18	1	1	1	1	-1	22.849±0.046	20.867±0.135	23.100±0.102
19	1	1	-1	1	1	23.046±0.139	22.545±0.131	23.049±0.064
20	1	-1	1	-1	-1	24.262±0.181	22.932±0.211	24.095±0.144
21	-1	-1	1	-1	1	26.542±0.013	22.776±0.156	25.572±0.122
22	1	1	1	-1	-1	24.973±0.057	21.268±0.259	24.614±0.194
23	0	0	0	-2.37	0	34.438±0.135	26.680±0.144	30.689±0.160
24	2.37	0	0	0	0	22.990±0.169	22.560±0.077	23.251±0.114
25	1	1	-1	-1	1	24.387±0.197	23.650±0.253	23.948±0.049
26	-1	-1	1	-1	-1	25.334±0.123	25.112±0.126	25.125±0.186
27	0	0	0	0	0	23.736±0.047	23.882±0.044	23.713±0.092
28	0	0	0	0	0	23.624±0.106	23.797±0.062	23.640±0.109
29	-1	-1	-1	-1	1	25.920±0.049	24.837±0.113	26.003±0.162
30	0	0	-2.3	0	0	/	/	/
31	-1	-1	-1	1	-1	24.118±0.116	23.288±0.220	23.258±0.137
32	-1	1	1	-1	-1	24.570±0.064	23.964±0.159	24.622±0.154
33	0	0	0	0	0	23.576±0.050	23.833±0.014	23.513±0.036
34	1	-1	-1	-1	-1	30.443±0.161	24.845±0.176	23.742±0.172
35	0	0	0	0	-2.37	23.914±0.088	23.421±0.147	23.631±0.076

36	0	0	0	0	0	23.757±0.177	23.559±0.146	23.561±0.111
37	0	2.378	0	0	0	23.388±0.069	23.469±0.056	23.317±0.083
38	-1	1	1	1	1	22.772±0.213	23.735±0.113	23.453±0.039
39	0	0	0	0	2.378	23.859±0.250	23.835±0.130	23.634±0.061
40	1	-1	1	1	1	23.642±0.094	24.323±0.094	23.697±0.055
41	-1	-1	-1	-1	-1	28.261±0.138	27.042±0.154	26.842±0.083
42	1	-1	-1	-1	1	25.242±0.274	25.571±0.081	24.324±0.430
43	0	0	2.37	0	0	24.040±0.102	24.182±0.164	23.993±0.136
44	-1	1	-1	1	1	24.834±0.191	23.230±0.124	23.206±0.134
45	-1	1	1	-1	1	25.724±0.226	25.412±0.124	24.555±0.095
46	0	0	0	2.378	0	23.855±0.036	23.757±0.083	23.769±0.144
47	1	1	1	-1	1	25.884±0.102	25.734±0.152	24.721±0.199
48	1	1	-1	1	-1	23.798±0.166	23.356±0.161	23.070±0.072
49	-1	-1	1	1	-1	23.743±0.063	23.788±0.153	23.797±0.103
50	-2.3	0	0	0	0	24.610±0.090	24.128±0.133	24.530±0.086

[a]A: primers, B: probe, C: DNA polymerase, D: Mg^{2+}, E: dNTPs.

[b]RSV、HMPV、INF are three virus used in this study.

[c]The results of Ct value in table are expressed as $\bar{\chi}$±s, and n=3.
