# Supplementary material for: Optimization of multiplex quantitative polymerase chain reaction based on response surface methodology and an artificial neural network-genetic algorithm approach
Source: PLoS One. 2018 Jul 25;13(7):e0200962. doi: 10.1371/journal.pone.0200962 (PMC6059488; doi:10.1371/journal.pone.0200962)
Supplement: S4 Table — (PDF) [file pone.0200962.s006.pdf]

S4 Table. Analysis of variance (ANOVA) for the Ct values of uniplex qPCR using RSM-CCD

Source	*Df* [b]	RSV[a]			INF[a]			HMPV[a]		
		SS[b]	*F*[b]	*P*	*SS*[b]	*F*[b]	*P*	*SS*[b]	*F*[b]	*P*
Model	20	36.280	10.620	<0.0001***	25.610	4.850	<0.0001***	14.710	4.840	<0.0001***
A	1	1.560	9.130	0.005**	0.022	0.084	0.774	3.010	19.780	0.0001**
B	1	2.720	15.94	0.0004**	3.010	11.400	0.002**	0.870	5.750	0.023*
C	1	0.056	0.330	0.573	0.019	0.073	0.789	0.950	6.26	0.018*
D	1	23.200	135.860	<0.0001***	11.400	43.16	<0.0001***	5.410	35.560	<0.0001***
E	1	0.690	4.030	0.0544	0.130	0.50	0.487	0.110	0.730	0.401
AB	1	0.035	0.200	0.6543	0.014	0.054	0.819	1.128×10^{-3}	7.421×10^{-3}	0.932
AC	1	4.805×10^{-4}	2.814×10^{-3}	0.9581	0.220	0.850	0.365	0.170	1.090	0.306
AD	1	0.340	2.010	0.167	1.880	7.110	0.013*	0.200	1.300	0.264
AE	1	0.130	0.750	0.395	0.094	0.350	0.556	0.027	0.180	0.674
BC	1	0.220	1.270	0.269	0.120	0.470	0.500	0.099	0.650	0.425
BD	1	5.281×10^{-4}	3.093×10^{-3}	0.956	0.400	1.530	0.227	0.029	0.190	0.665
BE	1	1.568×10^{-3}	9.183×10^{-3}	0.924	0.023	0.086	0.772	0.330	2.180	0.151
CD	1	0.069	0.400	0.531	0.320	1.210	0.281	0.17	1.100	0.304
CE	1	4.186×10^{-3}	0.025	0.877	1.060	4.030	0.055	0.10	0.660	0.422
DE	1	1.210	7.080	0.013*	0.220	0.830	0.371	0.17	1.140	0.296
A^2	1	0.160	0.940	0.341	0.160	0.620	0.438	2.675×10^{-3}	0.018	0.895

B^2	1	0.340	2.000	0.169	0.300	1.130	0.297	0.011	0.073	0.789
C^2	1	0.046	0.270	0.610	0.410	1.560	0.222	0.077	0.510	0.482
D^2	1	4.890	28.610	<0.0001***	5.700	21.570	<0.0001***	2.960	19.500	0.0001**
E^2	1	0.400	2.360	0.136	3.593×10^{-3}	0.014	0.908	0.018	0.120	0.733
Residual	28	4.780			7.400			4.260		
Lack of Fit	21	4.480	4.920	0.019*	6.590	2.720	0.089	4.060	6.740	0.008**
Pure Error	7	0.300			0.810			0.200		
Cor Total	48	41.070			33.000			18.970		
Adequate		15.923			12.187			11.686		

*p–value <0.05; **p–value <0.01; ***p–value <0.001.

[a]RSV、HMPV、INF are three virus used in this study.

[b]*Df*, Degree of freedom; *SS*, Sum of Squares; *F*, *F*-Value.
