# Supplementary material for: Optimization of multiplex quantitative polymerase chain reaction based on response surface methodology and an artificial neural network-genetic algorithm approach
Source: PLoS One. 2018 Jul 25;13(7):e0200962. doi: 10.1371/journal.pone.0200962 (PMC6059488; doi:10.1371/journal.pone.0200962)
Supplement: S5 Table — (PDF) [file pone.0200962.s007.pdf]

S5Table.Five-fold cross-validation of model IIfor uniplexqPCR

Neurons	RSV[a]		INF[a]		HMPV[a]	
	Error of fit（$\bar{\chi}$±s）	Error of prediction（$\bar{\chi}$±s）	Error of fit（$\bar{\chi}$±s）	Error of prediction（$\bar{\chi}$±s）	Error of fit（$\bar{\chi}$±s）	Error of prediction（$\bar{\chi}$±s）
1	0.041±0.037	0.037±0.053	0.070±0.024	0.031±0.045	0.046±0.046	0.025±0.032
2	0.027±0.052	0.031±0.041	0.040±0.056	0.036±0.071	0.023±0.061	0.022±0.034
3	0.018±0.043	0.021±0.034	0.025±0.045	0.035±0.054	0.018±0.043	0.017±0.027
4	0.016±0.042	0.021±0.047	0.025±0.037	0.028±0.048	0.017±0.054	0.017±0.024
5	0.017±0.027	0.014±0.022	0.026±0.025	0.026±0.033	0.018±0.037	0.019±0.026

[a]RSV、HMPV、INF are three virus used in this study.
