# Supplementary material for: Optimization of multiplex quantitative polymerase chain reaction based on response surface methodology and an artificial neural network-genetic algorithm approach
Source: PLoS One. 2018 Jul 25;13(7):e0200962. doi: 10.1371/journal.pone.0200962 (PMC6059488; doi:10.1371/journal.pone.0200962)
Supplement: S6 Table — (PDF) [file pone.0200962.s008.pdf]

S6Table.Performance numbers of BPNN-GA versus RSM models for uniplexqPCR

parameter	RSV[a]		INF[a]		HMPV[a]	
	Model I	Model II	Model I	Model II	Model I	Model II
R^2	0.884	0.950	0.776	0.934	0.776	0.899
MAE[b]	0.244	0.0004	0.285	0.0006	0.236	0.0003
MSE [b]	0.098	0.0012	0.151	0.0013	0.0870	0.0009

[a]RSV、HMPV、INF are three virus used in this study.

[b]MAE meansthe mean absolute error; MSE meansthe mean square error.
