# Supplementary material for: Optimization of multiplex quantitative polymerase chain reaction based on response surface methodology and an artificial neural network-genetic algorithm approach
Source: PLoS One. 2018 Jul 25;13(7):e0200962. doi: 10.1371/journal.pone.0200962 (PMC6059488; doi:10.1371/journal.pone.0200962)
Supplement: S7 Table — (PDF) [file pone.0200962.s009.pdf]

S7 Table. Optimization conditions and predictive Ct value of model I and model II for uniplex qPCR

Factors	RSV[b]		INF[b]		HMPV[b]	
	Model I	Model II	Model I	Model II	Model I	Model II
A[a](μM)	0.250	0.194	0.090	0.081	0.320	0.319
B[a](μM)	0.320	0.319	0.230	0.319	0.320	0.133
C[a](U[c]/μL)	0.120	0.164	0.120	0.181	0.020	0.081
D[a](mM)	2.830	0.319	3.200	0.283	2.320	0.319
E[a](mM)	0.320	0.319	0.130	0.157	0.320	0.082
Predictive Ct value	23.746	23.618	21.829	19.175	22.973	23.667

[a]A: primers, B: probe, C: DNA polymerase, D: Mg^{2+}, E: dNTPs.

[b]RSV、HMPV、INF are three virus used in this study.

[c]U: active unit of enzyme.
