# Supplementary material for: Optimization of multiplex quantitative polymerase chain reaction based on response surface methodology and an artificial neural network-genetic algorithm approach
Source: PLoS One. 2018 Jul 25;13(7):e0200962. doi: 10.1371/journal.pone.0200962 (PMC6059488; doi:10.1371/journal.pone.0200962)
Supplement: S8 Table — (PDF) [file pone.0200962.s010.pdf]

S8 Table. Optimal conditions for uniplex qPCR

Factors	RSV[b]	INF[b]	HMPV[b]
A[a](μmol/L)	0.250	0.090	0.320
B[a](μmol/L)	0.320	0.230	0.320
C[a](U[c]/μL)	0.120	0.120	0.020
D[a](mmol/L)	2.830	3.200	2.320
E[a](mmol/L)	0.320	0.130	0.320

[a]A: primers, B: probe, C: DNA polymerase, D: Mg^{2+}, E: dNTPs.

[b]RSV、HMPV、INF are three virus used in this study.

[c]U: active unit of enzyme
