# Supplementary material for: Optimization of multiplex quantitative polymerase chain reaction based on response surface methodology and an artificial neural network-genetic algorithm approach
Source: PLoS One. 2018 Jul 25;13(7):e0200962. doi: 10.1371/journal.pone.0200962 (PMC6059488; doi:10.1371/journal.pone.0200962)
Supplement: S1 Text — (PDF) [file pone.0200962.s011.pdf]

S1 Text. Results of uniplexqPCR

RSM

Statistical analysis results were analyzed using Design-Expert.V8.0.6 software. The effects of operating variables were investigated according tothe statistical analysis of CCD. Polynomial equations (1)-(3) represent the polynomial predictive model for uniplex qPCR for the three viruses (RSV, INF, and HMPV).

$$Y_1 = 25.550 - 0.190 \times A - 0.250 \times B - 0.730 \times D - 0.190 \times DE + 0.300 \times D^2 \quad (1)$$

$$Y_2 = 23.540 - 0.260 \times B - 0.510 \times D + 0.240 \times AD + 0.320 \times D^2 \quad (2)$$

$$Y_3 = 24.850 - 0.260 \times A - 0.140 \times B + 0.170 \times C - 0.350 \times D + 0.230 \times D^2 \quad (3)$$

In the polynomial equations, *Y* denotes the Ct value. A, B, C, D and E are the concentrations of primers, probes, DNA polymerase, $Mg^{2+}$and dNTPs, respectively. Coefficients that had no statistical significance are eliminated.

According to variance analysis (S1Table), the *F* and *P* values of the corresponding uniplex qPCR models for the three viruses were F=10.62 and P<0.0001, F=4.850 and P<0.0001, and F=14.840 and P<0.0001; the *P* values were all less than 0.05, indicating that there is evidence that at least one of the 5 predictors has an effect on the response.

BPNN-GA

The fitting error and prediction errorof five-fold cross-validation of model II for multiplex qPCR produced from training neurons in the hidden layer are provided in S2 Table. The fitting error and prediction error are the bases used to select the number of nodes in the hidden layer. When the two errors were relatively small, the

corresponding number of hidden-layer nodes was treated as the training model. Combining the fitting error and the prediction error, the number of hidden-layer nodes for RSV uniplexqPCR was 4, and 4 and 4 nodes were used for INF and HMPV, respectively.

RSM versus BPNN-GA

As shown in S3 Table. for uniplex PCR, the R^2 values of model II were closer to 1 than model I. The MAE and MSE values of model II were less than those of model I for all three viruses.

Validation

As indicated in S4 Table, the validated Ct value was 25.464 when using the optimized conditions for uniplex qPCR given by model I for RSV. Compared to the predictive Ct value of 23.746, the relative error was 6.748%. Meanwhile, the relative errors of the validated experimental Ct value versus the predictive value for INF and HMPV were 1.520% and 4.590% respectively, under the optimized conditions suggested by model I.
